# Ca^2+^ Flux: Searching for a Role in Efferocytosis of Apoptotic Cells in Atherosclerosis

**DOI:** 10.3390/jcm8122047

**Published:** 2019-11-21

**Authors:** Amir Tajbakhsh, Petri T. Kovanen, Mahdi Rezaee, Maciej Banach, Amirhossein Sahebkar

**Affiliations:** 1 Halal Research Center of IRI, FDA, Tehran, Iran; 2 Pharmaceutical Sciences Research Center, Shiraz University of Medical Sciences, Shiraz, Iran; 3 Wihuri Research Institute, 00290 Helsinki, Finland; 4 Department of Medical Biotechnology, School of Medicine, Mashhad University of Medical Sciences, Mashhad 9177948, Iran; 5 Department of Hypertension, WAM University Hospital in Lodz, Medical University of Lodz, Zeromskiego 113, 90-549 Lodz, Poland; 6 Polish Mother’s Memorial Hospital Research Institute (PMMHRI), 93-338 Lodz, Poland; 7 Biotechnology Research Center, Pharmaceutical Technology Institute, Mashhad University of Medical Sciences, Mashhad, Iran; 8 Neurogenic Inflammation Research Center, Mashhad University of Medical Sciences, Mashhad, Iran; 9 School of Pharmacy, Mashhad University of Medical Sciences, Mashhad 9177948, Iran

**Keywords:** atherosclerosis, Ca^2+^ homeostasis, calcification, calcium antagonists, cardiovascular diseases, efferocytosis, foam cells, macrophages, microRNA

## Abstract

In atherosclerosis, macrophages in the arterial wall ingest plasma lipoprotein-derived lipids and become lipid-filled foam cells with a limited lifespan. Thus, efficient removal of apoptotic foam cells by efferocytic macrophages is vital to preventing the dying foam cells from forming a large necrotic lipid core, which, otherwise, would render the atherosclerotic plaque vulnerable to rupture and would cause clinical complications. Ca^2+^ plays a role in macrophage migration, survival, and foam cell generation. Importantly, in efferocytic macrophages, Ca^2+^ induces actin polymerization, thereby promoting the formation of a phagocytic cup necessary for efferocytosis. Moreover, in the efferocytic macrophages, Ca^2+^ enhances the secretion of anti-inflammatory cytokines. Various Ca^2+^ antagonists have been seminal for the demonstration of the role of Ca^2+^ in the multiple steps of efferocytosis by macrophages. Moreover, in vitro and in vivo experiments and clinical investigations have revealed the capability of Ca^2+^ antagonists in attenuating the development of atherosclerotic plaques by interfering with the deposition of lipids in macrophages and by reducing plaque calcification. However, the regulation of cellular Ca^2+^ fluxes in the processes of efferocytic clearance of apoptotic foam cells and in the extracellular calcification in atherosclerosis remains unknown. Here, we attempted to unravel the molecular links between Ca^2+^ and efferocytosis in atherosclerosis and to evaluate cellular Ca^2+^ fluxes as potential treatment targets in atherosclerotic cardiovascular diseases.

## 1. Introduction

Cardiovascular diseases (CVDs) remain a leading cause of death and a significant cause of health loss in all regions of the world [1]. Atherosclerosis is the most common etiology of CVDs including ischemic heart disease due to coronary artery atherosclerosis, ischemic stroke due to carotid artery atherosclerosis, peripheral arterial disease usually due to atherosclerosis of the lower limb arteries, and many cases of heart failure [2]. Atherosclerosis is a dynamic, progressive, heterogeneous, chronic inflammatory and multifactorial disease that is affected by the interaction of environmental risk factors and the genetic background of an individual. Numerous studies have been able to define both environmental and genetic factors involved in the pathological events of atherogenesis [3,4,5].

In several genome-wide association studies, genetic risk factors for arterial calcification have been descibed such as *TCF7L2* and *WWOX* (in smoker) and *TNFRSF8* (in non-smoker) in patients with coronary artery calcification (CAC) [6]; *9p21*-rs4977574, *ADAMTS7-rs3825807*, and *PHACTR1-rs12526453* in patients with coronary artery disease (CAD) and myocardial infarction (MI) [7], *CDKN2A*, *CDKN2B-rs1333049*, *rs9349379-PHACTR1*, *MRAS*, *COL4A1/COL4A2*, and *SORT1* genes in MI [8]; *9p2-1rs16905644* and *PHACTR1* in patients with CAC [9]; *rs11353135-2q22.1*, *rs16879003-6p22.3*, rs5014012, rs58071836, rs10244825 on chromosome 7, *9q31.2-rs10918777, 16p13.3-rs13331874*, *18q12.1-rs4459623*, *13q32.1-rs6491315*, and *13q32-rs7492028* in patients with type 2 diabetes [10]. Interestingly, based on these studies, there is an overlap of risk genetic loci between arterial calcification and MI, indicating shared pathological components.

In this respect, a less-known factor related to the atherosclerotic plaque is the calcium ions (Ca^2+^), which play a vital role in various pathways of cellular metabolism, both in physiological and pathological states. Interestingly, variations both in intracellular and extracellular concentrations of Ca^2+^ have been reported to be involved in the generation of atherosclerotic lesions through different processes [11,12]. Extracellular mineralization of Ca^2+^ in the intimal layer of an atherosclerotic artery fundamentally influences both the architecture and the progression of a developing plaque and strongly contributes to the ultimate clinical outcome of atherosclerotic CVDs [11]. Regarding the role of intracellular Ca^2+^ in plaque formation/progression, it acts as a second messenger in macrophages, causing activation of downstream molecules and transcription of related genes. Accordingly, Ca^2+^ and Ca^2+^-related molecules and pathways involved in cellular Ca^2+^ signaling and homeostasis are associated with CVDs [12].

A generally accepted definition of efferocytosis is the clearance of apoptotic cells (ACs) by professional or nonprofessional phagocytic cells [13,14,15,16]. A large and ever-growing body of literature has demonstrated that the most important steps in the formation of atherosclerotic plaques are the accumulation of inflammatory cells (viz. macrophages and dendritic cells), foam cell formation, and defective efferocytic removal of ACs in the plaques [17,18]. Most importantly, defective efferocytosis of inflammatory apoptotic macrophages and macrophage foam cells results in their secondary necrosis and thereby contributes to the formation of an enlarged necrotic core, which weakens the plaque and makes it susceptible to rupture [19]. On the other hand, several studies have indicated that efficient removal of ACs by efferocytosis plays a protective role in atherogenesis by inhibiting the formation and progression of plaques, by preventing necrosis of plaques, and by reducing the inflammatory component inherent of atherosclerotic plaques [13,14].

**Figure 1:**
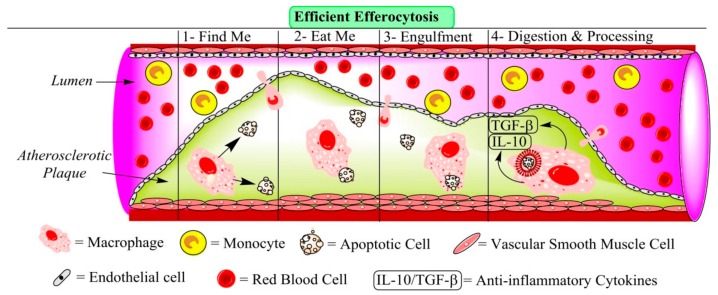
The 4 steps of efferocytosis are shown to take place in efferocytosis in an atherosclerotic plaque. In the plaque, one macrophage and two apoptotic cells (ACs) are shown. Generally, efferocytosis involves 4 main steps: 1. “Find-Me” signaling (sensing and migration of monocytes/macrophages toward the ACs); 2. “Eat-Me” (or “Don’t Eat-Me”) signaling (recognition/binding to the ACs); 3. engulfment; and 4. digestion and processing (degradation of the ingested cell with ensuing generation of cellular debris). Finally, the phagocyte shows an anti-inflammatory response by releasing IL-10 and TGF-β [14,23] (for more details, refer to Table 1). ACs release “Find-Me” signals that stimulate migration of phagocytes toward them. Moreover, ACs release “Tolerogenic and Keep-Out” signals to keep neutrophils and antigen-presenting cells away [24,25]. After “Find-Me” signaling, the phagocytes identify ACs via “Eat-Me” signals affected through ligands on ACs [14]. Following “Eat-Me” signaling, the phagocytic cells start engulfing ACs by cup formation and then digest and process the engulfed ACs. This step is necessary for the overall control of the efferocytic process, and it balances energy consumption after digestion. Moreover, it regulates the efferocyte enzyme activities, gene expression, expression of efferocytosis-related receptors, and further engulfment of ACs (multi-engulfment). Efferocytosis of cholesterol-filled macrophages in an atherosclerotic plaque prevents the development of an extracellular necrotic lipid composed of non-phagocytosed foam cells which have undergone secondary necrosis. Finally, the phagocytic cells begin to generate anti-inflammatory cytokines and regulators [14]. Altogether, efferocytosis is a very complex process and involves many regulatory pathways [14]. IL-10: interleukin-10; TGFβ: transforming growth factor beta.

**Figure 2:**
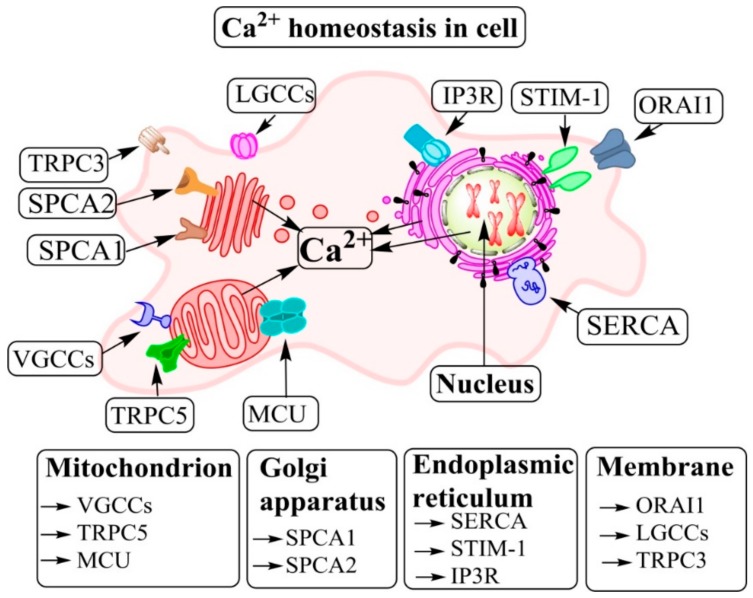
Cellular calcium [Ca^2+^] homeostasis and signaling: Organelles and molecules that are involved in Ca^2+^ pathways. There are several pathways which can change the amount of [Ca^2+^]_i_ and subsequently alter the biology of the cell. Several types of Ca^2+^-dependent proteins and subtypes in the cell include the store-operated Ca^2+^ channels (SOCs), Ca^2+^ transporters, transient receptor potential channels (TRPCs), voltage-gated Ca^2+^ channels (VGCCs), and ligand-gated Ca^2+^ channels [26]. Furthermore, phospholipase C (PLC) and inositol 1,4,5-trisphosphate and its receptor (IP3R), the stromal-interacting molecule (STIM), are located on the membranes of the sarcoplasmic/endoplasmic reticulum. Other subcellular organelles also have a role in [Ca^2+^]_i_ homeostasis of the cell, notably the mitochondrial Ca^2+^ uniporter (MCU) and the Ca^2+^/Mn^2+^ ATPases (SPCAs) secretory pathway of the Golgi [27,28,29]. Ca^2+^ enters the cytoplasm from the ER lumen and from the extracellular fluid via channels and pumps on the plasma membrane [30]. LGCCs: ligand-gated Ca^2+^-channels; ORAI: transient receptor potential canonical; SERCA: sarco/endoplasmic reticulum Ca^2+^ ATPase.

Efferocytosis is a cellular process that can occur continuously. Thus, a macrophage can clear several ACs rather than only a single AC, revealing the capacity of macrophages for continued clearance of ACs [14]. Efferocytosis includes several steps: the production of “Find-Me” signals such as lysophosphatidylcholine (LysoPC), Fractalkine (CX3CL1), and sphingosine-1-phosphate (S1P); the production of “Eat-Me” signals such as phosphatidylserine (PtdSer), milk fat globule-EGF factor 8 (MFG-E8), Mer tyrosine kinase (MerTK), Growth arrest-specific 6 (Gas6), and Protein S; engulfment such as ATP-binding cassette transporter A7 (ABCA7), interferon regulatory factor (IRF) 5 and 8, and peroxisome proliferator-activated receptor delta/gamma (PPAR)-δ/γ; and eventually secretion of anti-inflammatory cytokines (as post-engulfment) [13,14] (Table 1). For efferocytosis to work effectively, the many steps need to be performed in correct order. Among the factors orchestrating a well-functioning efferocytosis is activation of cell surface receptors of the phagocytes with ensuing increase in the cytosolic Ca^2+^ after their exposure to ACs. This event triggers a number of signaling pathways in the phagocytic cells, such as the macrophages, which are related to their stimulation, proliferation, and expression of relevant genes [20,21]. Moreover, managing the Ca^2+^ flux is crucial at post-engulfment steps of macrophages, leading to an appropriate anti-inflammatory response [22] (Figure 1 and Figure 2).

Several studies have identified that a defective Ca^2+^ flux with a diminishing effect on efferocytosis associates with augmented atherosclerosis [20,31,32,33]. Mechanistically, a defective Ca^2+^ flux in the phagocytes decreases the clearance of ACs and, moreover, attenuates the secretion of the anti-inflammatory cytokines interleukin-10 (IL-10) and the transforming growth factor beta (TGFβ), each of these three inhibitory effects being pro-atherogenic [22]. Furthermore, mutations in various genes linked to the Ca^2+^ flux can contribute to defective clearance of ACs [22,34,35]. In this review, we seek to clarify the underlying relationship between Ca^2+^ and efferocytosis in the atherosclerosis process and we also raise the possibility of Ca^2+^ being potentially a target for the treatment of atherosclerosis.

**Table 1:** The receptors/molecules that are suggested to be involved in efferocytosis.

Steps.	References	Molecules
Find-Me signal	[24,25,36]	LysoPC, ATP, P2Y_2_, ApoJ, ApoE4, Fractalkine (CX_3_CL1), S1P
Don’t Eat Me and Eat-Me signals		CD31, CD47, PtdSer, Caspase, MFG-E8, MerTK, Gas6, Protein S,
Engulfment and processing		TRPC3, ABCA7, IRF8, IRF5, PPAR-δ/γ, p38 MAPK activities, CDKN2B, TLR3, TRAF6, UCP2, Cathepsin G, Rac
Anti-inflammatory and tolerance responses		TGF-β, IL-10

Abbreviations: ABCA7: ATP-binding cassette transporter A7; ApoE: apolipoprotein E; ATP: adenosine triphosphate; CD: cluster differentiation; CDKN2B: cyclin-dependent kinase inhibitor 2B; Gas6: growth arrest-specific 6; IRF5: interferon regulatory factor 5; IRF8: interferon regulatory factor 8; LysoPC: lysophosphatidylcholine; MAPK: mitogen-activated kinase-like protein; MerTK: mer tyrosine kinase; MFG-E8: milk fat globule-EGF factor 8; PPAR-δ/γ: peroxisome proliferator-activated receptor *delta*/*gamma*; PtdSer: phosphatidylserine; Rac: a family small GTPase; S1P: sphingosine-1-phosphate; TLR3: toll-like receptor 3; TRAF6: tumor necrosis factor receptor-associated factor 6; TRPC3: transient receptor potential canonical 3; UCP2: uncoupling protein 2.

## 2. Regulatory Roles of Ca^2+^ in Cellular Functions

As an important secondary messenger, Ca^2+^ regulates a vast array of cellular functions [37]. Thus, the levels and fluxes of Ca^2+^ mediate receptor-mediated signal transduction, release, and activity of growth factors and proliferation, migration, and adhesion of cells [38,39]. The increase of intracellular calcium ([Ca^2+^]_i_) concentration (viz. cytoplasmic calcium ([Ca^2+^]_CYT_)) regulates various cellular processes, among them the activation of many enzymes. There are several pathways which can change the amount of [Ca^2+^]_i_ and subsequently alter the biology of the cell [22,40] (Figure 2).

In cells, Ca^2+^ is released from the endoplasmic reticulum (ER) (as the main store of [Ca^2+^]_i_), mitochondria, and Golgi and the influx of extracellular Ca^2+^ via channels and pumps on the plasma membrane is another source of [Ca^2+^]_i_ [30]. In addition, several types of Ca^2+^-dependent proteins and subtypes exist, such as the store-operated Ca^2+^ channels (SOCs), Ca^2+^ transporters, transient receptor potential (TRP) channels, voltage-gated Ca^2+^ channels (VGCCs), Ca^2+^ binding proteins (CBPs), and ligand-gated Ca^2+^ channels [26]. Furthermore, phospholipase C (PLC), inositol 1,4,5-trisphosphate (IP3), and the stromal-interacting molecule (STIM) on the membrane of the sarco/ER all play important roles in [Ca^2+^]_i_ homeostasis. In addition to ER, other organelles also have a role in [Ca^2+^]_i_ homeostasis of the cell, and they include the mitochondrial Ca^2+^ uniporter (MCU) and the Na^+^/Ca^2+^ exchanger (NCX) of the secretory pathway Ca^2+^/Mn^2+^ ATPases (SPCAs) of the Golgi [27,28,29,41]. Although the Golgi does not seem to be a place for Ca^2+^ storage, it has a critical role in the intracellular vesicular trafficking and secretion of proteins together with ER and Ca^2+^ [42]. It has been suggested that an increase of [Ca^2+^]_i_ may increase the secretion flow, which is a primary Golgi function and responsible for executing net unidirectional intracellular transport from the rough ER (RER) toward lysosomes and the cell surface [43].

Phair suggested that promotion of inter-Golgi transport may be responsible for extra protein delivery to secretory vesicles, especially together with translation accelerated in the RER, which could explain the excess collagen and elastin secretion by vascular smooth muscle cells (VSMCs) of the intimal layer of the arterial wall characteristic of atherogenesis [44]. According to this “calcium-atherogenesis” hypothesis, increased cytosolic Ca^2+^ in the VSMCs could be a partial explanation of the increased matrix protein secretion by these cells, leading to the growth of an atherosclerotic lesion.

## 3. The Role of Ca^2+^ in “Don’t Eat Me” Signalling

In effective efferocytosis, the engulfment of ACs and NCs by “Eat-Me” signals occurs together with downregulation of “Don’t-Eat-Me” signals such as CD47 and CD31 [45]. There is a growing body of literature that recognises the importance of CD47 in chronic disease [46,47]. Based on previously published papers, CD47 signaling regulates multiple distinct cellular processes such as efferocytosis, Ca^2+^ homeostasis, and nitric oxide (NO) [47,48]. CD47 regulates the Ca^2+^ influx into endothelial cells that is integrin dependent. In this case, thrombospondin-1/CD47 is a critical mediator of vascular cell Ca^2+^ levels [49]. Thrombospondin-1/CD47 signaling positively modulates cytoplasmic Ca^2+^ [50]. In this respect, B6H12, as a function-blocking antibody for thrombospondin-1 binding, prevented Ca^2+^ influx triggered via an integrin ligand [50]. Furthermore, cross-linking CD47 leads to intracellular Ca^2+^ mobilization on the surface of brain endothelial cells, enhanced permeability and promotion of src kinase activity, and phosphoinositide-3-kinase–protein kinase B (PI3K/AKT1) activity in vivo [51].

Decreased blood flow secondary to peripheral vascular disease underlies many chronic diseases in elderly people, and importantly, secretion of thrombospondin-1 by vascular cells is upregulated after injury and in chronic wounds, typical of peripheral arterial disease in the elderly [49]. This particularly applies to diabetes, in which thrombospondin-1 mediates delay in re-endothelialization following arterial injury [52]. Many studies have demonstrated that blood vessel diameter is reduced and that blood flow is limited due to the ability of thrombospondin-1 to inhibit vascular responses to NO [53]. Physiologically, the formation of NO by endothelial cells triggers relaxation of the contractile VSMC layer of blood vessels and, for the prevention of vasoconstriction via increased VSMC contractility, also reduces the accessibility of Ca^2+^ in the VSMCs [54]. Available evidence shows that balanced interaction and NO signaling are defective in CVD. One explanatory factor for such defect is the upregulation of thrombospondin-1 and CD 47 in CVD [49]. Moreover, thrombospondin-1/CD47 signaling triggers the production of enzymatic reactive oxygen species (ROS), which induces inflammation and endothelial dysfunction with ensuing further reduction of blood flow and, via endorgan ischemia, increases the severity of vascular disease. Indeed, a key molecular mechanism behind the such deleterious effects are the thrombospondin-1/CD47 signaling-dependent changes in Ca^2+^ flux, which prevent the activation and production of endothelial NOS [49].

More recently, the therapeutic potential of anti-CD47 antibodies has been demonstrated in atherosclerotic CVD in experimental animals. Such beneficial effects of the CD47-blocking antibodies may decrease the burden of CVD via restoring impaired efferocytosis in the atherosclerotic lesions [55,56]. Moreover, as discussed above, in endothelial cells, thrombospondin-1 binding to CD47 leads to a decrease in NO production by eNOs and thereby results in vasoconstriction [55]. Since the vasoconstrictive effect of the thrombospondin-1/CD47 signaling appears to be enhanced during aging, agents that attenuate the thrombospondin-1/CD47-dependent vasoregulation could be of value when treating vascular problems that result from aging [46,56]. Enhanced CD47 also leads to defective phagocytic clearance via macrophages on the surface of dying cardiomyocytes in vitro. Following myocardial ischemia and reperfusion, inhibition of integrin-associated protein CD47 with blocking antibodies increased the clearance of dead myocytes by cardiac phagocytes and increased the resolution of cardiac inflammation [47].

## 4. Scavenger Receptors and Efferocytosis in Atherosclerotic Plaques

Scavenger receptors (SRs) are a family of surface-expressed receptors, which are involved in the internalization of extracellular components and targets them to lysosomal compartments [57]. Regarding atherosclerosis, the SRs are the key molecules implicated in the recognition, uptake, and processing of modified lipoproteins, notably of the oxidatively modified low-density lipoproteins (Ox-LDL) [57,58]. Importantly, several studies have revealed that the activity of SRs in the phagocytic process depends on the influx of [Ca^2+^]_e_ [58,59,60]. Also, Ox-LDL stimulates the rise in [Ca^2+^]_i_, which leads to foam cell formation through the CD36 pathway and assembly of F-actin, as shown in cultured U937-derived macrophages [61]. Interestingly, the CD36-specific oxidized phospholipids in Ox-LDL induce CD36-dependent activation of Vav proteins, which, again, trigger CD36-mediated macrophage foam cell formation via Ca^2+^ and dynamin 2-dependent processes [62]. Thus, the Vav-dynamin signaling axis plays a critical role by generating a Ca^2+^ signal in macrophages exposed to CD36-specific oxidized phospholipids. CD36 is only one of the many SRs implicated to play a crucial role in atherosclerosis, and it also mediates efferocytosis [59,63]. Lectin-like oxidized LDL receptor 1 (LOX-1) is another scavenger receptor that mediates vascular responses to Ox-LDL and that is involved in the pathogenesis of atherosclerosis including monocyte adhesion, foam cell formation, apoptosis, proliferation, migration of smooth muscle cells (SMCs), and plaque instability [64]. Mechanistically, the human LOX-1 recognizes Ca^2+^ dependently on a PtdSer molecule on the cell surface of an AC molecule, the maximal recognition taking place at millimolar levels of Ca^2+^ [65].

Based on the above information about the important role of Ca^2+^ in the scavenger receptor-dependent atherogenesis, it is feasible to assume that Ca^2+^ antagonists could be useful as potential anti-atherosclerotic reagents. This assumption is supported by the finding demonstrating that, in macrophages derived from mice in which the Vav protein-dependent Ca^2+^ flux was genetically absent, i.e., in macrophages with impaired Ca^2+^ signaling, the uptake of Ox-LDL by CD36 and ensuing foam cell formation are reduced [66]. Moreover, it is of great interest that Ca^2+^ antagonists have been shown to slow experimental atherosclerosis in animals and to suppress the generation of new lesions and the progression of existing lesions in humans [67].

Taken together, alteration in the activity of SRs strongly regulates macrophage foam cell formation, efferocytosis activity, and thereby the development of atherosclerotic plaques. Unraveling the molecular mechanisms in these processes have allowed defining molecular targets for pharmacotherapy of atherosclerosis regarding the Ca^2+^ metabolism in macrophages. Moreover, the Ca^2+^ channel blocker nifedipine inhibits Ox-LDL-induced apoptosis of endothelial cells by downregulating LOX-1 in the endothelial cells [68]. Since endothelial apoptosis is a root cause of endothelial erosion, which, again, may lead to acute coronary syndromes and MI [69,70], Ca^2+^ antagonists may have the potential to prevent the progression of atherosclerotic lesions at multiple levels.

## 5. The Vital Role of Ca^2+^ in the Efferocytic Engulfment Process

As discussed above, Ca^2+^ is an essential ion in the regulation of cell biology and homeostasis. Moreover, resolution of inflammation is a vital aspect of homeostasis especially during atherosclerosis [26]. Furthermore, as an active process that leads to resolution of inflammation, efferocytosis is regulated by several molecular pathways. In this line, Ca^2+^ is involved in some critical steps of the efferocytosis also related to atherosclerosis. After recognition of AC, these steps include polymerization of actin and phagocytic cup formation during engulfment, which are critical for the uptake and digestion of ACs and subsequent anti-inflammatory cytokine production [22,35,71,72,73,74]. For example, for engulfment of ACs by phagocytes, Ca^2+^ flux is essential already during recognition of ACs [22]. Moreover, Ca^2+^ is involved in some of the vital steps during engulfment of ACs, such as actin enrichment and polymerization adjacent to the AC, formation of a phagocytic cup, and secretion of anti-inflammatory cytokines, notably TGFβ [22]. In this regard, downregulation via RNA interference (RNAi) of *stim-1* or *jph-1*, two genes involved in extracellular Ca^2+^ influx in *C. elegans*, leads to actin reorganization and inhibition of phagocytosis of ACs [22]. In this line, it has been suggested that an increase in [Ca^2+^]_i_ through AC recognition leads to the alteration of the actin cytoskeleton required for AC engulfment [23,35,75]. Indeed, after the initiation of phagocytosis, activation of the small GTPase Rac1 and remodeling of the actin cytoskeleton are required, as they increase the movement of phagocyte membrane to engulf the target AC [22].

It is well known that the polymerization of the filamentous actin, the F-actin, is vital for the formation of the phagocytic cup in phagocytosis and efferocytosis [76]. F-actin is regulated via the proteins of the Rho family of GTPases, of which Rac1, Cdc42, and RhoA are recognized to coordinate the dynamics of F-actin [77,78,79]. Rac1 activation is critical in the formation of the phagocytic cup by the regulation of actin polymerization [80]. Moreover, Rac1 activation is needed for lamellipodia formation [78]. After AC recognition, Rac activation is facilitated via activation of the guanine nucleotide exchange factors (Rac-GEFs). In this line, Elmo1 and Dock, as Rac-GEFs, are important for AC uptake and for cell migration in mammalian efferocytosis [23,81,82]. In contrast, RhoA stimulation triggers actomyosin contraction and the stabilization of F-actin, and it is needed for the stress fiber formation [78]. RhoA-GTP has been indicated to prevent phagocytosis of ACs [83]. The overexpression of RhoA negatively regulates AC engulfment by Rho-associated coiled-coil-containing protein kinase (ROCK) [84]. Moreover, the activity of ROCK kinase by myosin light chain phosphorylation is able to trigger the accumulation of actomyosin and cellular contraction [85,86]. Accordingly, crosstalk between RhoA and Rac leads to a successful engulfment in efferocytosis [86]. Furthermore, Cdc42 activation increases the formation of short actin filaments, and it is also needed for filopodia formation [78,87]. Similarly to Rac, Cdc42 also enhances the engulfment of ACs [87].

It was reported that the use of 1,2-bis(*o*-aminophenoxy)ethane-N,N,N′,N′-tetraacetic acid (BAPTA) as a Ca^2+^-chelating agent and cytochalasin D as an inhibitor of actin polymerization inhibited phagocytic cup formation and actin polymerization during phagocytosis in *C. elegans* [22]. Interestingly, Gronski et al. indicated that both [Ca^2+^]i and [Ca^2+^]e stores appear to be necessary for TGFβ secretion by macrophages, which, by inhibition of Ca^2+^ release, results in reduced TGFβ production [22]. The association between efferocytosis and Ca^2+^ shown in different studies will be discussed in the next section.

## 6. Role of Ca^2+^ Flux and Macrophage Metabolism in Efferocytosis of Apoptotic Cells in Atherosclerotic Plaques

As mentioned above, foam cells in the arterial intima are derived from macrophages exposed to an excess of modified lipoproteins, particularly of Ox-LDL, which is both proinflammatory and pro-atherogenic [88]. The formation of foam cells via accumulation of Ox-LDL leads to a reduction of efferocytosis by a decrease in phagocytosis and production of anti-inflammatory cytokines in the atherosclerotic plaque [73]. Thus, an increase of monocyte differentiation into macrophages and uptake of Ox-LDL eventually leads to a decrease in AC engulfment and efferocytosis by macrophages [89,90]. Moreover, Ox-LDL may result in foam cell formation through increased [Ca^2+^]_i_ that occurs in various cell types such as VSMCs and macrophages [91,92,93]. This enhanced [Ca^2+^]_i_ is provided by the [Ca^2+^]_i_ store (including the ER store) and production of IP3. Additionally, when added to cultured Jurkat cells, to fibroblasts, to endothelial cells, or to cells belonging to the U937 monocyte-macrophage cell line, Ox-LDL increases the intracellular level of the ROS, and the resulting oxidative stress then activates the Ca^2+^-calcineurin pathway of the transcription factor NFAT (Nuclear Factor of Activated T cells) [92,93]. Ox-LDL and the peroxidized lipids contained in it rapidly increased the intracellular concentration of Ca^2+^. Importantly, trapping of the intracellular Ca^2+^ with ethylene glycol tetraacetic acid (EGTA) or BAPTA abolished the Ox-LDL-induced NFAT activity, and moreover, preincubation with thapsigargin, as an ER Ca^2+^-ATPase inhibitor, inhibited the increase of [Ca^2+^] induced by Ox-LDL in the T lymphocyte cell line Jurkat and the human microvascular endothelial cells [92,93], indicating the involvement of Ca^2+^ in this particular effect of Ox-LDL. Since NFAT mediates some proinflammatory effects of T cells, like secretion of IL2 and tumor necrosis factor-alpha (TNF-α), upregulation of the surface receptor CD40L, and activation of the transcription factor nuclear factor kappa B (NFκB), the Ca^2+^-dependent NFAT activation by Ox-LDL can be considered a contributor to the inflammatory process in atherosclerotic lesions [92]. Furthermore, there are several signaling pathways stimulated via oxLDLs that can prompt apoptosis and that subsequently increase atherosclerosis [94,95]. A study by Vindis et al. demonstrated that apoptosis stimulated by Ox-LDL was mediated via two separate Ca^2+^-dependent mitochondrial pathways, one mediated by Ca^2+^/calpain/mitochondrial permeability transition pore/cytochrome C/caspase-3 and the other mediated by a factor, which is cyclosporine-insensitive and caspase independent [93].

## 7. Fundamental Role of Ca^2+^ Flux in Phagocytic Cells and in the Anti-Inflammatory Response to Engulfment of ACs in Efferocytosis and Atherosclerosis

The transient receptor potential canonical (TRPC) channels, as nonselective cation channels which can function either as SOCs or as receptor-operated channels (ROCs), appear to be important players in CVDs, particularly in diabetic atherosclerosis [96,97]. The role of TRPC3 is verified in macrophage survival and efferocytosis in atherosclerotic lesions [20]. Transient receptor potential vanilloid type 2 (TRPV2) and transient receptor potential melastatin 2 (TRPM2) seem to be important for proper phagocytosis, and as a result, they may be associated with efferocytosis of ACs in atherosclerotic lesions. The *TRPC3*^−/−^ macrophages showed impaired efferocytosis activity compared to *TRPC3^+/+^* macrophages [72]. TRPC3 plays a role in two vital steps in the expansion of atherosclerotic plaques, notably in the survival of macrophages via two pathways including PI3K/AKT and NFκB and in the clearance process of ACs [20]. In this respect, TRPC3 on the membrane of macrophages is vital for their survival and also for an efficient efferocytosis by them. Accordingly, the diminished survival signaling promoted apoptosis and defective clearance of ACs by the TRPC3-deficient macrophages in mice [72]. In the study by Tano et al., it was reported that the nonregulated, constitutive Ca^2+^ influx could increase cell survival and could reduce apoptosis by the PI3K/AKT and NF*κ*B pathways via a mechanism related to calmodulin/calmodulin kinase II (CAM/CAMKII) [98]. Therefore, it seems that the CAM/CAMKII axis could connect functional Ca^2+^ influx and stimulation of cell survival processes.

## 8. Relations between ER, Ca^2+^, and Efferocytosis in Atherosclerosis

ER stress is involved in atherosclerosis and is engaged in the development of the atherosclerotic plaques [31,32]. Most significantly to the pathogenesis of atherosclerosis, macrophages display ER stress after being exposed to lipotoxic signals which are known to be present in atherosclerotic plaques [33]. The stress-related alterations in ER increase both macrophage apoptosis and inflammation, both of which lead to decreases in macrophage efferocytosis and to enhanced development of the atherosclerotic plaque [99,100]. Based on previous research, increased apoptosis has been shown to be associated with unstable angina, ruptured plaques, and atherothrombotic events, linking the pathobiology of the plaque with CVDs of major clinical importance [101,102].

**Figure 3:**
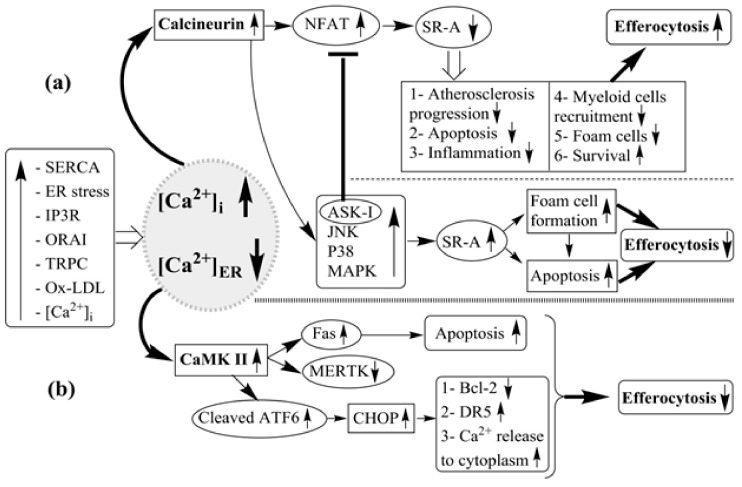
Molecular pathways linking endoplasmic reticulum stress and subcellular Ca^2+^ movements to efferocytosis in atherosclerosis: Effects of upregulation of sarcoplasmic reticulum Ca^2+^-ATPase (SERCA), endoplasmic reticulum (ER) stress, inositol 1,4,5-trisphosphate receptor (IP3R), ORAI, transient receptor potential canonical (TRPC), oxidized low density lipoprotein (Ox-LDL), and [Ca^2+^]_i_ on the calcineurin-mediated and calmodulin kinase II (CaMKII)-mediated cellular pathways associated enhanced or diminished efferocytosis. (**a**) The Orai1-activated increase of [Ca^2+^]_i_ stimulates calcineurin, which results in triggering of two signaling pathways including calcineurin–apoptosis signal-regulating kinase 1 (ASK1)-p38/JNK and calcineurin-nuclear factor of activated T cells (NFAT) [108]. Mitogen-activated protein kinases (MAPKs), c-Jun N-terminal kinase (JNK), and p38 MAP have critical roles in the modulation of class A scavenger receptors (SR-A) expression [108]. The NFAT transcription factors act as negative modulators of SR-A expression [109]. A signaling pathway, which positively modulates the SR-A expression and stimulates foam cell formation in vitro, is calcineurin–ASK1-p38/JNK signaling pathway, while the calcineurin–NFAT pathway is a negative regulator of SR-A expression [109]. Activation of ASK1 via a negative feedback mechanism restricts the inhibitory influence of NFAT on the expression of SR-A [109]. (**b**) The released Ca^2+^ triggers CaMKII, which leads to activation of apoptotic pathways. Especially, CaMKII has many roles including the signal transducer and activator of transcription 1 (STAT1) activation, triggering the proapoptotic signal transducer and stimulation of the FAS death receptor by JNK [71,105]. ER stress leads to an increase in [Ca^2+^]_Cyt_, which in turn prompts FAS death receptor expression via the CaMKIIγ and JNK pathways in vivo [71]. CaMKII knockdown enhances macrophage efferocytosis receptor tyrosine-protein kinase MER (MerTK) expression, which in turn increases efferocytosis, and, in atherosclerotic lesions, should ultimately result in smaller necrotic cores. The increase of ATF6 leads to an enhanced efferocytosis through the expression of MerTK and in the absence of CaMKIIγ in macrophages [110]. Cleavage of ATF6 leads to an increase of C/EBPα-homolog protein (CHOP). Increase in the CHOP promotes cytosolic Ca^2+^ in the macrophages via CHOP-mediated stimulation of oxidase ERO1α in the ER, which in turn triggers Ca^2+^ release from the IP3R channel in the membranes of ER and also in an increased expression of the death receptor-5 (DR5) and in decreased expression of the death receptor-5 (Bcl-2), a mediator of cell survival [104,111]. These alterations result in increased cellular apoptosis and in reduced efferocytosis, a combination which is particularly pro-atherogenic.

ER is the main Ca^2+^ storage site in cells, and together with the Golgi apparatus, the Ca^2+^ content of ER is critical for appropriate protein folding and transport [103]. An excessive burden of unfolded proteins in the ER lumen leads to ER stress and activates signal transduction pathways, collectively termed the unfolded protein response (UPR) [103]. When exposed to the lipotoxic signals associated with atherosclerosis, macrophages show lipid-chaperone (FABP4)-mediated ER stress, UPR, and lipotoxic death [33]. On the other hand, reducing such lipid-induced metabolic ER stress signaling in macrophages protects hyperlipidemic mice from atherosclerosis [33]. Several molecules are involved in the UPR in ER, like the luminal Ca^2+^, which can change the protein folding in the ER and can lead to ER stress by misfolded proteins [103]. Increase in the expression of C/EBPα-homolog protein (CHOP), an effector molecule in the UPR, promotes cytosolic Ca^2+^ in the macrophages via CHOP-mediated stimulation of oxidase ERO1α in the ER, which in turn triggers Ca^2+^ release from the inositol 1,4,5-trisphosphate receptor (IP3R) channel in the membranes of ER [104]. Moreover, the released Ca^2+^ triggers CaMKII, which leads to the activation of apoptotic pathways. Especially, CaMKII has many roles including signal transducer and activator of transcription 1 (STAT1) activation, triggering of the proapoptotic signal transducer, stimulation of the First Apoptosis Signal (FAS) death receptor through the c-Jun N-terminal kinase (JNK), release of cytochrome c from the mitochondria, and stimulation of nicotinamide adenine dinucleotide 3-phosphate (NADPH) oxidase-mediated ROS [105] (Figure 3). Besides apoptosis, CaMKII is involved in the impaired inflammation resolution in atherosclerotic plaques [106]. CaMKII increases AMP-activated kinase (AMPK)-activated protein kinase 2 (MK2)-mediated 5-lipoxygenase (5-LOX) phosphorylation by increasing Ser271 phosphorylation and translocation to the nuclear membrane [107]. In this regard, resolvin D1 (RvD1) reduces the 5-LOX phosphorylation and the nuclear localization via suppressing the CaMKII activation, and therefore, it limits biosynthesis of the proinflammatory leukotriene B4 (LTB4) [107].

Furthermore, the [Ca^2+^]_i_ of macrophages is increased via inhibition of the sarco/endoplasmic reticulum Ca^2+^ ATPase (SERCA), which pumps Ca^2+^ from the cytosol to the ER lumen [112,113]. Li et al. found that cholesterol loading of macrophages with ensuing accumulation of unesterified cholesterol in the normally cholesterol-poor ER membranes leads to rigidity of the membranes with ensuing inhibition of SERCA2b, the form of SERCA found in macrophages [114]. Consequently, such cholesterol overload of the ER membrane results in the depletion of ER Ca^2+^ stores. The authors hypothesized that the excess unesterified cholesterol in macrophages of advanced atherosclerotic lesions leads to loss of function of SERCA2b due to its decreased conformational freedom in the cholesterol-enriched rigid ER membranes. The authors also suggested that this biophysical model could be used to explain the critical connection between the excess of unesterified cholesterol, UPR induction, ER Ca^2+^ depletion, and apoptotic death of macrophages.

## 9. The Relation Between Ca^2+^/Calmodulin-Dependent Protein Kinase II Gamma (CaMKIIγ) and ER Stress in Atherosclerosis

Cellular apoptosis mediated by ER stress is involved in several pathological conditions; for example, ER stress leads to release of Ca^2+^ from the ER with ensuing increase in [Ca^2+^]_Cyt_, which in turn prompts FAS death receptor expression via CaMKIIγ and the JNK pathway both in vitro and in vivo [71] (Figure 3). Activation of CaMKIIγ in macrophages plays a crucial role in the development of necrotic thin-capped plaques and advanced atherosclerotic lesions in humans and mice [110]. Thus, in *Ldlr*^−/−^ mice, the atherosclerotic lesions containing CaMKIIγ-deficient macrophages had smaller necrotic cores and showed evidence of enhanced macrophage efferocytosis, which was associated with increased expression of the macrophage efferocytosis receptor tyrosine-protein kinase MER (MerTK). Furthermore, the defective CaMKIIγ macrophages showed enhanced expression of the activating transcription factor 6 (ATF6), which is an important activator of the ER stress response and prompts liver X receptor-α (LXRα), a transcription factor induced by Mertk. These findings suggested that the CaMKIIγ/ATF6/LXRα/MerTK pathway in the macrophages is critical for the progression of the atherosclerotic plaques [110]. Also, Gas6 and protein S (on phagocytes) are important molecules in the efferocytic process as “Eat-Me” signals, which bind to PtdSer on ACs to intensify efferocytosis [115,116]. They are associated with MertK on macrophages and cause an increase of efferocytosis [116,117]. Therefore, in advanced atherosclerosis, the use of CaMKII antagonists could be valuable in inhibiting apoptosis and inflammation since they would not act like immune-suppressors, which act via ER stress.

## 10. The Relation Between Mitochondrial Ca^2+^ and Efferocytosis in Atherosclerosis

The mitochondria are critical for cell survival and in the initiation of cell death [118]. The mitochondrial Ca^2+^ level, albeit much lower than that in the ER and Golgi, influences the susceptibility to cell death activation [119]. Ca^2+^ channels including a voltage-dependent anion-selective channel (VDAC) and MCU participate in the mitochondrial Ca^2+^ uptake, and they were proposed as potential regulators of cell death [118]. MCU provides Ca^2+^ transport into the mitochondrial matrix without energy consumption [120]. Interestingly, CaMKIIγ is also involved in apoptosis through mitochondrial cytochrome c release and loss of the mitochondrial membrane potential in the mitochondrion. Furthermore, CaMKII was found to increase Ca^2+^ uptake by mitochondria and thereby to lead to increased apoptosis in vitro and potentially to an ineffective efferocytosis and thereby to plaque progression [71].

## 11. Roles of Mitochondria and AMP-Activated Protein Kinase (AMPK) in Efferocytosis in Atherosclerosis

Several studies have demonstrated that the clearance of ACs is vital for the resolution of tissue inflammation and the maintenance of tissue homeostasis [13,14,121,122,123]. It is suggested that mitochondrial AMPK, as a regulator of cellular metabolism, also promotes clearance of the ACs [123]. In macrophages, the AMPK is immediately activated after exposure to ACs or lysoPC on the surface of ACs. LysoPC is a specific phospholipid which is produced and released from ACs as a “Find-Me” signal for efferocytosis by phagocytes [123]. In the phagocytes, stress and/or increased energy demand results in increased oxygen consumption and in an increased content of ROS in the mitochondria. These conditions depend on the mobilization of intracellular Ca^2+^ into the mitochondria of phagocytes and are important for efficient efferocytosis [123]. It was suggested that mitochondria act as key organelles in ROS generation and Ca^2+^-associated damage in cardiac ischemia-reperfusion [124]. AMPK activation promotes the synthesis of ATP and of microtubules and chemokinesis and provides energy during the recognition and AC engulfment (for multi-engulfment of ACs). Jiang et al. showed an increased uptake of ACs in lungs of mice that had received lysoPC [123]. These results offer an association between lysoPC, as a marker of ACs, and uptake of Ca^2+^ by mitochondria, as a suggested marker for engulfment of ACs. Additionally, Jiang and coworkers showed that the prevention of AMPK production by dexamethasone reduces the clearance of apoptotic thymocytes in vitro and in mice [123].

## 12. Mitochondrial Fission and Related Factors in Efferocytosis and Atherosclerosis

The process of AC engulfment as a critical step in the efferocytosis consists of several important functions related to atherosclerosis including seeking, recognizing, and ingesting ACs and maintaining cellular homeostasis of the phagocytes after the engulfment. Therefore, efficient clearance depends on the capacity of phagocytes, especially macrophages, to ingest multiple ACs. Accordingly, the factors that influence the continued clearance of ACs by phagocytes are vital for efficient efferocytosis [121]. Degradation of the ingested AC and vesicular trafficking in the phagocyte are essential for engulfing another AC [125]. Mitochondrial fission facilitates appropriately digestion of AC-derived debris and vesicular trafficking, both of which are Ca^2+^-dependent in mice [125]. Therefore, through mitochondrial fission, macrophages can clear additional ACs in response to AC uptake, a capacity which is necessary for an apoptotic microenvironment in vivo [125].

**Figure 4:**
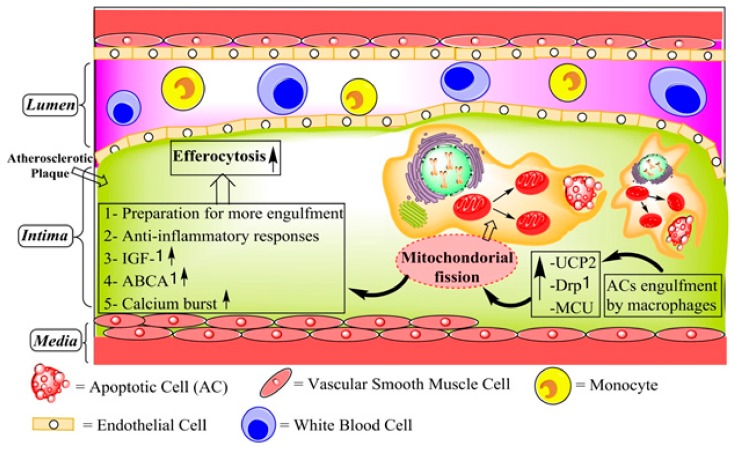
The role of mitochondrial fission and related factors in efferocytosis and atherosclerosis: The mitochondrial fission is associated with efferocytosis via Ca^2+^ response and is mediated via the dynamin-related protein 1 (Drp1) [35]. Engulfment of ACs alters mitochondrial membrane potential in phagocytic cells. Mitochondrial membrane uncoupling protein 2 (UCP2) has been found to be an important regulator of efferocytosis in phagocytic cells [121]. Mitochondrial fission associates with increases of ATP-binding cassette transporter A1 (ABCA1) and of insulin-like growth factor-1 (IGF-1). IGF-1 is an antiapoptotic factor which inhibits calcification and increases the growth of vascular smooth muscle cells [130].

The mitochondrial fission is associated with efferocytosis via Ca^2+^ response and is mediated by dynamin-related protein 1 (DRP1), a member of the cytosolic GTPase family involved in the uptake of multiple ACs in the macrophages [35] (Figure 4). In addition to DRP1, MCU is also involved in this process, and its downregulation results in mitochondria-mediated defective efferocytosis in the *Ldlr*^−/−^/*Drp1*^−/−^ mice. These findings were proven by dexamethasone as an inducer of thymocyte apoptosis and resulted in defective efferocytosis [125]. Park et al. have shown that the mitochondrial membrane uncoupling protein 2 (UCP2) was upregulated in phagocytes engulfing ACs [126]. The UCP2, a mitochondrial membrane carrier protein, is located in the mitochondrial inner membrane and reduces the mitochondrial membrane potential [126]. Engulfment of ACs alters mitochondrial membrane potential in phagocytic cells, and interestingly, UCP2 was also found to be an important regulator of efferocytosis in phagocytic cells [121]. Thus, lack of UCP2 expression resulted in impaired engulfment of ACs, whereas UCP2 overexpression increased efferocytosis in UCP2-deficient mice. The deficiency of UCP2 led to an accumulation of ACs in atherosclerotic plaques, to an enhancement of necrotic cores in the plaques and accelerated atherosclerosis in *Ucp2*^−/−^ mice [127]. DRP1 is a mediator of mitochondrial fission and is involved in apoptosis [128]. It has been demonstrated that DRP1-UCP2 pathways maintain the efferocytic ability of phagocytic cells and that DRP1-deficient macrophages are defective in efferocytosis both in vitro and in vivo [129]. The role of UCP2 in clearance of ACs provides some additional insights toward an understanding of the role of this molecule in the complex processes taking place in advanced atherogenesis [121].

## 13. Tumor Necrosis Factor Receptor-Associated Factor 6 (TRAF6) in Efferocytosis and Atherosclerosis

TRAF6 is located in the cytoplasm and interacts with cell surface receptors like CD40 (a marker of the proinflammatory M1 macrophage phenotype), which is a member of the tumor necrosis factor (TNF) receptor family and plays a role in the progression of atherosclerosis [131]. TRAF6 is involved in atherosclerosis by inducing inflammatory mediators, and it is associated with excessive inflammation in atherosclerosis-related diseases. Activation of CD40, as a TNF receptor, induces the formation of the TRAF6-p62 complex and subsequently increases NF*κ*B signaling and thereby decreases the production of anti-inflammatory cytokines and efferocytosis in macrophages [132]. Lack of myeloid TRAF6, but not of endothelial TRAF6, transcriptionally activates NF*κ*B and so leads to increased ER stress and impaired expression of anti-inflammatory cytokines like IL-10 in a mouse model of atherosclerosis [133]. Thus, the myeloid *Traf6*^−/−^ genotype increased susceptibility to apoptosis induced by Ox-LDL and also reduced the clearance capacity of phagocytic cells in the *Traf6*^−/−^/*ApoE*^−/−^ mice [133]. A study by Lutgens et al. demonstrated that CD40-TRAF6-defective mice had reduced numbers of circulating proinflammatory Ly6C^high^ monocytes and increased numbers of the anti-inflammatory M2 macrophages in atherosclerotic plaques [134], suggesting the CD40-TRAF6 interaction as a potential target for the development of novel therapeutics.

## 14. Interferon Regulatory Factor 8 (IRF8) and Factor 5 (IRF5) in Efferocytosis and Atherosclerosis

One member of the interferon (IFN) regulatory factor family, IRF8, also known as “interferon consensus sequence binding protein”) is expressed in hematopoietic cells [135]. It has been shown that defective IRF8 accelerates atherosclerotic lesion formation in *IRF8*^−/−^/*ApoE*^−/−^ mice [136]. In this mouse model, the *IRF8*^−/−^ macrophages displayed reduced expression of CD36, diminished efferocytosis, reduced IL-10 release, and decreased lipid uptake. Importantly, *IRF8* codes for a transcription factor of the IRF family that is known to be important in monocyte differentiation [74]. Along these lines, Crosslin et al. performed genome-wide association analyses in humans to investigate genetic variants related to circulating monocyte count, a biological variable associated with atherosclerotic plaque formation [137]. They identified a common variation in IRF8 (a molecule also regulating monocyte differentiation), which significantly associated with monocyte counts in the circulation.

Another member of the IRF family is IRF5, which plays a role in the generation of the pro-inflammatory M1 macrophages [138], which were shown by Seneviratne et al. to possess the reduced capacity of efferocytosis [139]. Importantly, Seneviratne et al. also demonstrated that IRF5 deficiency leads to a decrease of CD11c^+^ macrophages and to an increase of CD11c^−^ macrophages in atherosclerotic lesions of *ApoE*^−/−^/*Irf5*^−/−^ mice, of which the latter ones enhance integrin-β3 and its ligand (MFGE8) expression and promote efferocytosis [139]. Genetic variants of the *IRF-5* gene, like the 5 bp indel (insertion/deletion) (CGGGG) polymorphism, might be associated with susceptibility to plaque vulnerability and rupture and with ensuing acute coronary syndromes [140]. Hall and Wei commented on recent findings indicating that suppression of IRF5 via siRNA in the infarcted myocardium of *ApoE*^−/−^ mice reduced M1 polarization while reprogramming macrophage polarization toward the M2 phenotype could not be validated during the short observation period, during which the expression levels of IL-10 and TGF-β remained unchanged [141]. Finally, IRF5 plays an important role in activating IL-23/IL-12 and TNF-α involved in the responses of T helper 1 (Th1) and T helper-17 (Th17), which can react to these cytokines by inducing macrophages to adopt the M1 phenotype [142,143].

## 15. The Role of ORAI1 Store as a Part of the Operated Ca^2+^ Channel in Efferocytosis Related to Atherosclerosis

Agonist activation of G-protein-coupled receptors in the plasma membrane of mammalian cells activates phospholipase C with ensuing generation of the second messenger inositol 1,4,5 triphosphate (IP3), which then activates the IP3 receptor in the ER membranes and causes discharge of ER-stored Ca^2+^ [144]. The ER-anchored sensor protein stromal interaction molecule 1 (STIM1) senses the depletion of ER Ca^2+^ stores and then redistributes to the ER-plasma membrane apposition sites, where it recruits ORAI1, a subunit of the Ca^2+^-release-activated channels (CRAC) to allow Ca^2+^ to enter the cell through a CRAC channel, which is the main path for Ca^2+^ entry into non-excitable cells in the mammalian body [145,146].

ORAI1 is involved in several critical functions related to atherosclerosis, as shown in the elegant study by Liang et al. [109]. Thus, ORAI1is necessary for the Ox-LDL-prompted Ca^2+^ influx in cultured macrophages, which then leads to foam cell formation. Importantly, ORAI1 expression was increased in atherosclerotic plaques of *ApoE*^−/−^ mice, and moreover, knockdown of *Orai1* for 4 weeks in the cholesterol-fed *ApoE*^−/−^ mice attenuated atherosclerosis progression by preventing apoptosis of lesional macrophages, by decreasing the expression of inflammatory genes in the atherosclerotic lesions, and by attenuating recruitment of myeloid cells into the atherosclerotic plaques [109].

Increase of [Ca^2+^]_i_ leads to stimulation of Ca^2+^/calmodulin-dependent kinases pathways [147]. The ORAI1-activated increase of [Ca^2+^]_i_ stimulates calcineurin, which results in triggering of two signaling pathways including calcineurin–apoptosis signal-regulating kinase 1 (ASK1)-p38/JNK and calcineurin–NFAT [108] (Figure 3). The MAPKs, JNK, and p38 MAP have critical roles in the modulation of SR-A expression [108]. The calcineurin–ASK1-p38/JNK signaling pathway positively modulates the SR-A expression and stimulates foam cell formation in vitro, while the calcineurin–NFAT pathway is a negative regulator of SR-A expression [109]. Activation of ASK1 via a negative feedback mechanism restricts the inhibitory influence of NFAT on the expression of SR-A [109]. Collectively, ORAI1 signaling results in the expression of some inflammatory genes and recruitment of immune cells, such as macrophages, T lymphocytes, and neutrophils in atherosclerotic lesions [109].

## 16. MicroRNAs and Their Effects on Efferocytosis Via Ca^2+^

Inadequate proliferation, migration, and apoptosis of VSMCs are pathological processes implicated in atherosclerosis. Thus, their proliferation and migration are increasing the size of the early lesions, while their apoptosis is thinning the fibrous cap of advanced lesions. Previous studies have reported that microRNAs (miRs) play crucial roles in the regulation of VSMC proliferation and migration. In this respect, it was found that hsa-miR-148b is significantly downregulated in human atherosclerotic carotid plaques and that restoration of the function of hsa-miR-148b in cultured VSMCs transfected with a hsa-miR-148b mimic markedly prevented their proliferation and migration, with heat shock protein 90 (HSP90) being a direct target of the hsa-miR-148b in the VSMCs [148]. Furthermore, the expression of the hsa-miR-148b was negatively associated with the HSP90 mRNA levels in the atherosclerotic carotid plaques of the patients. Regarding the hsa-miR-148b effect, it inhibited HSP90 expression by binding to the 3′-untranslated region (UTR) of its messenger RNA (mRNA), while upregulation of HSP90 abolished the hsa-miR-148b-mediated prevention of proliferation and migration of VSMCs. Importantly, it has been also found that HSP90, as a ubiquitous molecular chaperone, is overexpressed in the inflammatory regions of unstable human atherosclerotic plaques with thin caps and that the small-molecular inhibitors of HSP90 reduce the inflammatory response in atherosclerotic lesions in mice [149]. Finally, given that hsa-miR-148b acted as an antiproliferative and antimigratory agent via targeting HSP90 in VSMCs, either of these two molecules could potentially be a novel therapeutic target in atherosclerosis [148,150].

## 17. Therapeutic Aspects

Maintenance of an efficient efferocytosis in advancing atherosclerotic plaques is potentially of considerable clinical importance. This conclusion stems from the knowledge that, without being ingested by neighboring vital macrophages, old macrophage foam cells die, they trigger an inflammatory response, and their bodies build up to form an ever-growing necrotic lipid core, which ultimately renders the plaque susceptible to rupture and cause even fatal atherothrombotic complications. Therefore, understanding cellular Ca^2+^ metabolism and the related molecules/pathways which are involved in the generation of foam cells and in decreased efferocytic engulfment of ACs are of great importance when considering to attenuate the progression of atherosclerotic lesions [18]. In this regard, Ca^2+^ plays a physiological and anti-atherosclerotic role by promoting the migration and survival of macrophages, and by enhancing the secretion of anti-inflammatory cytokines by them. Importantly, both the release of Ca^2+^ from the ER and the entry of extracellular Ca^2+^ via CRAC channels into the phagocytes are crucial for the engulfment of an AC and the anti-inflammatory signalling induced in phagocytes by contact with ACs [22]. Consequently, a defective Ca^2+^ flux in the phagocytes decreases the clearance of ACs and the secretion of the anti-inflammatory cytokines IL-10 and TGFβ.

In view of the above, it is somewhat surprising and apparently paradoxical that in vitro, in vivo, and clinical studies have revealed that administration of Ca^2+^ antagonists, i.e., Ca^2+^ channel blockers, can potentially inhibit or retard the development of atherosclerotic plaques (see Table 2). This apparent paradox can be understood, at least in part, when one considers that the evolutionary old and well-conserved acute and sustained Ca^2+^ fluxes within phagocytes are involved also in the uptake, metabolism, and deposition of lipids in phagocytes, which are crucial elements of atherosclerosis, an evolutionary young disease of civilization. Moreover, Ca^2+^ antagonists have been found to prevent calcification of coronary atherosclerotic plaques, an effect likely based on multiple local anti-atherosclerotic actions which are mechanistically different from their ability to counteract vasoconstriction and vasospasm. Interestingly, azithromycin therapy of patients with chronic obstructive pulmonary disease did improve the ability of alveolar macrophages derived from the patients to phagocytose ACs ex vivo [151]. Moreover, lovastatin, a 3-hydroxy-3-methylglutaryl coenzyme A (HMG-CoA) reductase inhibitor, when given to patients with chronic obstructive pulmonary disease increased efferocytosis of apoptotic neutrophils and T cells ex vivo by monocyte-derived macrophages and alveolar macrophages isolated from the patients [151,152].

In addition, anti-inflammatory therapeutics may restore an attenuated efferocytic signaling and so may lead to enhanced efferocytosis. For example, docosahexaenoic acid (DHA), an omega-3 fatty acid, promotes resolution of inflammation by facilitating efferocytosis through polarization of macrophages toward the anti-inflammatory M2 phenotype [122]. DHA also has pro-resolving effects mediated via PPAR-γ activation, which results in increased efferocytosis [122]. Maresin 1 (MaR1), produced by endogenous DHA of macrophages, is involved in tissue homeostasis, tissue regeneration, and host defense, and it controls pain with the resolution of local acute inflammation in vivo and in vitro [166,167]. 5-aminoimidazole-4-carboxamide-1-β-D-ribofuranoside (AICAR), a cell-permeable adenosine analog, could be used to treat inflammatory conditions caused by impaired macrophage clearance, as it increases activation of the p38 MAPK (p38MAPK) and thereby promotes phagocytosis of ACs by macrophages [168]. Interestingly, it was demonstrated that RGD-AnxA5, a variant of annexin A5, which targets cell surface phosphatidylserine (PS), transfers AnxA5 from an inhibitor into a stimulator of efferocytosis and so enhances the engulfment of ACs [169]. Moreover, RGD-AnxA5 did augment the secretion of IL-10 during efferocytosis in vivo, thereby possibly adding to an anti-inflammatory local environment. Finally, annexin A1/lipocortin 1-mimetic peptide nanoparticles containing the anti-inflammatory peptide Ac2-26 decreased polymorphonuclear neutrophil (PMN) recruitment and blunted inflammation in a self-limited zymosan-induced peritonitis model [170].

Recently, several studies have shown that synthetic ACs, as examples of non-cell-based therapies, might be used to enhance efferocytosis. An example of such synthetic ACs are the PtdSer-decorated nanoparticles which mimic ACs [171,172]. Hosseini et al. showed in apoE-KO mice that PtdSer liposomes, which mimic ACs, effectively reduce atherosclerotic lesions by inducing poly-reactive IgM producing B1a lymphocytes [173]. The atherosclerosis-inhibiting effect associated with decreased numbers of CD8^+^ and CD4^+^ T cells, decreased expression of the adhesion molecule VCAM-1; of the chemoattractant MCP-1; and of the cytokines IL-1β, IL-12, IL-18, and TNF-α and increased the expression of TGF-β in the atherosclerotic lesions. According to these studies, PS nanoparticles as non-cell-based therapies could be utilized as potential treatments for atherosclerosis. It has been demonstrated that there is no specific toxicity in AC-based therapies. Indeed, in a phase I/IIa clinical trial, a single intravenous infusion of donor mononuclear early ACs into patients with leukemia was found to be safe and effective in preventing graft-versus-host disease after allogeneic hematopoietic cell transplantation [174].

Findings from studies with Ca^2+^-channel blockers such as diltiazem, verapamil, and nifedipine have supported the notion that the regulation of cellular Ca^2+^ fluxes is relevant when attempting to slow the progression of atherosclerotic lesions [175]. Thus, prevention of L-type Ca^2+^ channels by nifedipine in patients with CAD has led to increased endothelial function and, subsequently, also decreased, though not significantly, the atherosclerotic plaque size in patients with CAD [165]. Moreover, several clinical trials have investigated directly or indirectly the effects of some intervention, related to Ca^2+^, for the treatment of atherosclerosis, as summarized in Table 2 [155,157,158,161,164,165]. For example, in coronary patients undergoing percutaneous intervention, treatment with nifedipine (30–60 mg/day), on top of a statin, for 24 months improved coronary endothelial function [165]. When compared with placebo, the levels of high-density lipoprotein (HDL) cholesterol were higher on nifedipine and, as expected, the blood pressure levels were lower. Moreover, in the most constricting segment, nifedipine reduced vasoconstriction in response to acetylcholine while nifedipine was not found to affect the volume of atherosclerotic plaques during the study period. 

Matsumoto et al. investigated the administration three oral doses of atreleuton (25 mg, 50 mg, or 100 mg), a lipoxygenase inhibitor, in patients with a recent acute coronary syndrome and found that the use of this drug slowed plaque development [158]. A study by Lee et al. examined the effects of sarpogrelate, a selective 5-HT_2A_ receptor antagonist, at a dose of 300 mg/day combined with aspirin 100 mg/day or aspirin 100 mg/day alone for 6 months in 40 diabetic patients with 10–75% coronary artery stenosis [157]. The results of this study showed no significant change in severity of CAD when assessed by determining the Ca^2+^ score, maximal stenosis, and plaque volume (calcified vs. noncalcified). The volumes of plaques were reduced in the “sarpogrelate + aspirin” group compared to the aspirin monotherapy group. Thus, sarpogrelate treatment may decrease coronary artery plaque volume, mainly the noncalcified portion, in patients with diabetes [157].

Statins are a class of drugs which inhibit the activity of 3-hydroxy-3-methyl-glutaryl coenzyme A reductase, the rate-controlling enzyme in the pathway of cholesterol biosynthesis [176]. They increase the activity of hepatic low-density lipoprotein receptors and thereby hepatic uptake of circulating LDL-particles, which lower the concentration of plasma LDL-cholesterol [177] and successfully decrease CVD, MI, and ischemic stroke [18,178,179]. Statins are divided in two main groups, hydrophilic statins such as fluvastatin, rosuvastatin, and pravastatin and lipophilic statins such as atorvastatin as well as simvastatin [18]. Interestingly, of the statins, both hydrophilic statins (fluvastatin and pravastatin) and a lipophilic statin (atorvastatin) have been found to inhibit inorganic phosphate-induced apoptosis and the related calcification of culture VSMCs via the Gas6-mediated survival pathway [180,181]. However, the pathogenesis of atherosclerosis-related coronary calcification is complex and involves particularly extracellular calcification. Such calcification is initiated by extracellular microcalcification, which is promoted by calcification of microvesicles and exosomes secreted by VSMCs of synthetic phenotype typical of atherosclerotic lesions [182]. However, as indicated in Table 2, statins may also result in an increase of coronary Ca^2+^ compared with other interventions [160,162,163,183]. In clinical studies, coronary calcification is measured by determining the coronary artery calcium score or “CAC score” or by evaluating the “% dense calcium volumes of plaques” by virtual histology-intravascular ultrasound. In a study by Kwon et al., after a 12-month statin therapy, a positive association was found between the reductions in the volumes of the necrotic cores and dense Ca^2+^ in 218 patients with stable coronary plaques [156]. Patients with the strongest reduction of high-sensitivity C-reactive protein (hsCRP) also had the greatest decrease in necrotic core volume, while those with the smallest hsCRP reduction had larger necrotic cores and enhanced dense Ca^2+^ volumes. Moreover, no significant associations between the changes in the levels of LDL-cholesterol and alterations in the necrotic core or dense Ca^2+^ volumes were observed. Thus, in this study, a relationship was found between the anti-inflammatory action, but not the LDL-cholesterol lowering effect, of a 12-month statin therapy and plaque stabilization characterized by three compositional changes: reduction of the necrotic core, thickening of the fibrous cap, and reduction of the volume of dense Ca^2+^.

Although the coronary Ca^2+^ score, i.e., the degree of coronary calcification, associates with the severity of CAD [184], paradoxically, as indicated in Table 2, several clinical trials have now revealed that statins, the mainstream drugs to prevent and treat CAD, may even enhance the Ca^2+^ score in atherosclerosis [160,162,163]. However, there are several, at least apparently controversial points, which are discussed in the relevant literature. A number of studies have indicated that the CAC score is a risk factor for atherothrombotic events [185,186]. Future myocardial events are predicted by an annual increase in CAC > 100 units as well as CAC > 15% [186,187]. However, it is well established that treatment with a statin decreases CAD events via reduction in LDL levels [188]. Furthermore, the statins lead to stabilization of coronary artery plaques [189]. Indeed, the statin-dependent increase in the calcification of atheroscleotic an atherosclerotic plaque may be the mechanisim that leads to stabilizing effects in vivo [190]. In this case, statins were reported to stabilize atherosclerotic plaques via reducing lipid-rich and the materials of necrotic plaque and, in parallel, enhancing atherosclerotic plaque calcification [190,191].

However, in studies with large population sizes, it was observed that statins increase CAC [192,193,194]. In this respect, it seems that high-dose statin therapy results in stabilizing of plaques with a concomitant increase in the volume of plaque calcification. Obviously, then, the effects of statins on coronary plaque calcification may be manifold both in terms of changes in the quality and/or quantity of the calcification. Moreover, as the actions of statins on atherosclerotic plaques are mostly indirect, i.e., they depend on the LDL-lowering effect of statins, and to some extent direct (pleiotropic) effects of the circulating statin, the observed changes in the plaque calcification likely result from a multitude of local metabolic modifications within the plaques. The degree of the lowering effects of LDL-cholesterol may regulate coronary vascular calcification [187]. In contrast, statins are also indicated to protect against the calcification of coronary vascular [195,196]. Achenbach et al. suggested that cerivastatin treatment decreases further development of calcification in patients with coronary calcifications and with LDL cholesterol levels >130 mg/dL [195]. Lee et al. suggested that statin treatment enhances the level of HDL cholesterol, resulting in reduction of the CAC score [183]. They also suggested that HDL cholesterol, as a mediator, may have an inhibitory regulating function in the progression of CAC and statins [183]. Lee et al. also observed that the association of statins and CAC is more complicated and that other mechanisms may be involved, such as lipid-independent mechanisms (e.g., cathepsin signaling) [183,197,198]. Further investigations are needed to fully establish the found association of cathepsin and vascular calcification process by statins. However, today, it has become clear that statins could lead to and increase CAC as an untoward or perhaps even benificial side effect as a component of a “statin paradox”, i.e., statins increase calcium in atheromas even when they shrink them [192,193]. Ikegami et al. indicated that the annual rate of CAC with a protein convertase subtilisin/kexin type 9 (PCSK9) inhibitor and a statin therapy (14.3%), as a combination therapy, is lower than monotherapy with statin (29.7%) [187]. Thus, it appears that, when used as a combination therapy with statins, the PCSK9 inhibitors offer a possible option to reduce or even inhibit CAC development. 

A study by Kalia et al. indicated that adherence to lipid-lowering therapy was increased in patients who underwent electron beam tomography (EBT) for the visualization of coronary Ca^2+^ [199]. Indeed, high CAC scores were strongly related to good adherence to statin therapy. Accordingly, the authors recommended EBT coronary Ca^2+^ measurement, since it may enhance the motivation of asymptomatic patients to modify also their lifestyle.

Raggi et al. demonstrated in a large group of patients on statin treatment and having the same levels of plasma LDL-cholesterol that, in patients with continued CAC progression, the risk of first MI was 17-fold higher than in patients without CAC progression [200]. Consequently, these controversial results raise questions about the direct and indirect effects on vascular calcification of the use of statin and also lipid-dependent or -independent mechanisms. As a result, it is possible that some of the drugs that have an effect on atherosclerotic lesions and the inflammatory components of the lesions also modulate the cellular Ca^2+^ fluxes and subsequently lead to an enhanced efferocytosis in the atherosclerosis plaques [201].

## 18. Conclusions

Ca^2+^ regulates numerous cellular processes and pathways that are critically related to atherosclerosis, one of such pathways being efferocytosis by macrophages. Indeed, Ca^2+^ is involved in all steps of efferocytosis including migration of macrophages, survival of macrophages, actin polymerization, formation of a phagocytic cup during AC engulfment, intracellular processing of ingested ACs, and secretion of anti-inflammatory cytokines. Importantly, considerable evidence shows that Ca^2+^ antagonists also possess anti-atherogenic properties. Treatments based on Ca^2+^, such as those with the CAMKII inhibitors that increase the resolution of inflammation in the atherosclerotic plaque, could function as anti-inflammatory therapies without having an immunosuppressive effect. To reach this goal, it is essential to recognize the critical factors responsible for ineffective efferocytosis by macrophages in atherosclerotic lesions. When considering the ability of some drugs currently in clinical use, e.g., statins and azithromycin, in partially correcting defective efferocytosis of lung alveolar macrophages in patients suffering from lung disease, the challenge remains to develop drugs targeted to activate macrophages in the atherosclerotic arterial lesions to more effectively accomplish their role as efferocytic cells and to thereby slow down the progression of atherosclerosis. Hopefully, a better understanding of the basic biology of cellular Ca^2+^ metabolism in a complex pathophysiological setting at the tissue level, like an inflamed lipid-rich atherosclerotic lesion, will aid us when attempting to design personalized and targeted pharmacotherapies in patients with atherosclerotic CVDs.

## Figures and Tables

**Table 2:** Clinical trials with drugs and agents having potential effects on both Ca^2+^ and atherosclerosis.

Author and Year	Disorders	Intervention	Numbers of Patients	Effect on Plaque Calcification	Effect on Plaque Volume
Bruining et al., 2009 [153]	Coronary artery disease	Perindopril (angiotensin-converting enzyme inhibitors)	118 patients	↓	↓
Magnani et al., 1992 [154]	Atherosclerosis and plaque evolution, with hypertension	Verapamil, dihydropyridines and diphenylalkylamines (calcium antagonists)	550 patients	↓	↓
Zanchetti et al., 2002 [155]	Asymptomatic carotid atherosclerosis	Lacidipine (calcium antagonist)	2334 patients	↓	↓
Kwon et al., 2017 [156]	Coronary artery disease	Rosuvastatin (HMG-CoA reductase inhibitor)	218 patients	↑	↓
Lee et al., 2017 [157]	Diabetic patients with 10–75% coronary artery stenosis	Sarpogrelate (5-HT2A receptor antagonist)	40 patients	↓	↓
Matsumoto et al., 2017 [158]	Patients with recent acute coronary syndrome	Atreleuton (5-lipoxygenase inhibitor VIA-2291)	54 patients	↓	↓
Park et al., 2016 [159]	Statin and atheroma vulnerability evaluation study	Rosuvastatin (HMG-CoA reductase inhibitor)	225 patients	Unchanged	↓
Koskinas et al., 2016 [160]	ST-elevation myocardial infarction	Rosuvastatin (HMG-CoA reductase inhibitor	44 patients	↑	↓
Matsumoto et al., 2016 [161]	Metabolic syndrome	Aged garlic extract	27 patients	↓	↓
Puri et al., 2014 [162]	Coronary atheroma	Rosuvastatin vs. atorvastatin (HMG-CoA reductase inhibitors)	71 patients	↑	↓
Eshtehardi et al., 2012 [163]	Moderate coronary artery disease	Atorvastatin (HMG-CoA reductase)	20 patients	↑	↓
Kojima et al., 2011 [164]	Hypertension	Azelnidipine and amlodipine (calcium channel blockers)	199 patients	↓	↓
Lüscher et al., 2009 [165]	Coronary artery disease	Nifedipine (calcium channel blocker)	454 patients	↓	↓

**Abbreviations**. HMG-CoA: 3-hydroxy-3-methylglutaryl coenzyme A; 5-HT2A: Serotonin 2A. ↑: enhancing effect; ↓: reducing effect.

## Abbreviations

ABCA7: ATP-binding cassette transporter A7; ACAT: acyl-CoA:cholesterol acyltransferase; ACs: apoptotic cells; AICAR: 5-aminoimidazole-4-carboxamide-1-β-D-ribofurano-side; AMPK: AMP-activated kinase; *ApoE*^−/−^: apolipoprotein E knockout; ASK1: apoptosis signal–regulating kinase 1; ATF6: activating transcription factor 6; Ca^2+^: calcium; [Ca^2+^]_CYT_: cytoplasmic calcium; [Ca^2+^]_i_: intracellular calcium; CAC: coronary artery calcium; CAM/CAMKII: calmodulin/calmodulin kinase II; CaMKIIγ: Ca^2+^/calmodulin-dependent protein kinase II gamma; CBPs: Ca^2+^-binding proteins; CDKN2B: cyclin-dependent kinase inhibitor 2B; CHOP: C/EBPα-homolog protein; CRAC: calcium-release-activated channel; CVD: cardiovascular disease; DC; dendritic cell; DHA: docosahexaenoic acid; Drp1: dynamin-related protein 1; ER: endoplasmic reticulum; Gas6: growth arrest-specific protein 6; hsCRP: high-sensitivity C-reactive protein; HMG-CoA: hydroxymethylglutaryl-CoA; HSP90: heat shock protein 90; IL-10: interleukin-10; IP3: inositol 1,4,5-trisphosphate; IP3R; inositol 1,4,5-trisphosphate receptor; IRF5: interferon regulatory factor 5; IRF8: interferon regulatory factor 8; JNK: c-Jun N-terminal kinase; LOX-1: lectin-like oxidized LDL receptor 1; 5-LOX: 5-lipoxygenase; LTB4: leukotriene B4; LysoPC: lysophosphatidylcholine; MAPK: mitogen-activated protein kinases; MaR1: maresin 1; MCU: mitochondrial Ca^2+^ uniporter; MerTK: receptor tyrosine-protein kinase MER; MI: myocardial Infarction; MiR: microRNA; MFG-E8: milk fat globule-EGF factor 8; MK2: MAPK-activated protein kinase 2; mRNA: messenger RNA; NADPH: nicotinamide adenine dinucleotide 3-phosphate; NCX: Na^+^/Ca^2+^ exchanger; NFAT: nuclear factor of activated T cells; NFκB: nuclear factor kappa B; NO: nitric oxide; ORAI1: calcium release-activated calcium modulator 1; Ox-LDL: oxidized low-density lipoprotein; PI3K: phosphatidylinositol-3-kinase; PPAR: peroxisome proliferator-activated receptor; PLC: phospholipase C; PMN: polymorphonuclear neutrophil; PtdSer: phosphatidylserine; Rac1: Rac Family Small GTPase 1; RER: rough ER; RNAi: RNA interference; ROCs: receptor-operated Ca^2+^ channels; ROS: reactive oxygen species; RvD1: resolvin D1; ROCK: Rho-associated coiled-coil-containing protein kinase; S1P: sphingosine-1-phosphate; Selk: selenoprotein K; SERCA: sarco/endoplasmic reticulum Ca^2+^ transport ATPase; SOC: store-operated Ca^2+^ channel; SPCA: secretory pathway Ca^2+^/Mn2+ ATPase; SR: scavenger receptor; STAT1: signal transducer and activator of transcription 1; STIM: stromal-interacting molecule; TGFβ: transforming growth factor beta; Th1: T helper 1; Th17: T helper 17; TNF-α: tumor necrosis factor-alpha; TRAF6: tumor necrosis factor receptor–associated factor 6; TRP channels: transient receptor potential channels; TRPC: transient receptor potential canonical; TRPM2: transient receptor potential melastatin 2; TRPV2: transient receptor potential vanilloid type 2; UCP2: mitochondrial membrane uncoupling protein 2; UPR: unfolded protein response; UTR: 3′-untranslated region; VDAC: voltage-dependent anion-selective channel; VGCC: voltage-gated Ca^2+^ channel; VSMC: vascular smooth muscle cell.
